# Effectiveness of dose-escalated topiramate monotherapy and add-on therapy in neurosurgery-related epilepsy: A prospective study

**DOI:** 10.1097/MD.0000000000023771

**Published:** 2020-12-24

**Authors:** Yu-Tse Liu, Guo-Tai Chen, Yin-Cheng Huang, Jih-Tsun Ho, Cheng-Chi Lee, Cheng-Chia Tsai, Chen-Nen Chang

**Affiliations:** a Department of Neurosurgery, Chang Gung Memorial Hospital, Linkou Branch and school of medicine; b Department of Neurosurgery, Chang Gung Memorial Hospital, Kaohsiung Branch and school of medicine; c Department of Neurosurgery, Chang Gung Memorial Hospital, Chiayi Branch and school of medicine, Chang Gung University, Taiwan.

**Keywords:** topiramate, seizures, neurosurgical-related epilepsy, monotherapy, add-on therapy, dose-escalation

## Abstract

**Background:**

Lesional and symptomatic causes of epilepsy are the most common neurological disorders of the brain. Topiramate effectively controls newly diagnosed epilepsy and refractory focal seizures, but high-dose topiramate does not improve seizure control. This study aimed to evaluate the clinical efficacy and safety of dose-escalated topiramate as first-line monotherapy and add-on therapy in patients with neurosurgery-related epilepsy.

**Material and Methods:**

A total of 55 neurosurgical patients with epilepsy were divided into monotherapy and add-on therapy groups and both groups received topiramate via the dose-escalation method. The primary efficacy outcomes were seizure-free rate and seizure response rate. Adverse events and seizure frequency were recorded.

**Results:**

The seizure response rate in the first month of monotherapy was significantly better than that of add-on therapy (89% vs 65%, *P* < .05), but no significant differences were found in seizure response rates between the 2 groups after 2 months of treatment. Both monotherapy and add-on therapy were effective in controlling seizures, with mean seizure frequency of 0.725 vs 0.536 and seizure-free rate of 88% vs 78.6%. Both treatments showed good improvement of seizure frequency in patients without tumor. The efficacy of monotherapy was better than that of add-on therapy (80% vs 29.2%) in patients with body mass index (BMI) ≤24. However, add-on therapy was better than monotherapy (76.7% vs 21.4%) in patients with BMI > 24. Dizziness (25.5%) and headache (16.4%) were the most common adverse events. No severe adverse event such as cognitive impairment was observed.

**Conclusions:**

Dose-escalated topiramate monotherapy and add-on therapy demonstrate good efficacy and safety, with fewer adverse events in seizure control in neurosurgical patients.

## 1 Introduction

Epilepsy, a central nervous system disorder, is usually characterized by unpredictable and recurrent seizures that affect a variety of patients mental and physical functions.^[1]^ About 50 million people worldwide have epilepsy, making it one of the most common neurological diseases globally.^[2]^ The prevalence of epilepsy in the US (Minnesota), UK, India, South Korea and China are 6.8, 5.3, 4.7, 2.41, and 1.8 per 1000 adults, respectively.^[2]^ Although the etiology of most epilepsy is unclear, head injuries due to traumatic brain injury, stroke and brain tumors are the common causes of epilepsy currently.^[3,4]^ In addition, patients with head injuries often require neurosurgery, which results in the loss of neurons and disruption of communication between neurons in the brain and subsequent neurosurgery-related epilepsy.^[1,4]^

Anti-epileptic drug (AED) therapy is the main treatment for most patients with epilepsy, and its ultimate goal is to prevent seizures completely.^[4,5]^ In current clinical practice, the use of a single seizure medication can control seizures in about 50% to 60% of patients with epilepsy, and adding a second seizure medication can control an additional 10% of seizures.^[6]^ The remaining 30% with refractory epilepsy cannot be controlled with medications alone.^[5,7]^ Recent studies further show that combination therapy is associated with better seizure-free rates than monotherapy, especially in patients with drug-resistant epilepsy.^[3,8]^ On the other hand, in clinical practice, titration to the maximum tolerated dose is the accepted principle of antiepileptic therapy. Nearly half of patients receiving the maximum tolerated dose of anti-epileptic drugs will achieve effective control of seizures within 1 year.^[9]^

Topiramate, a sulfamate-substituted monosaccharide (2,3:4, 5-bis-O-(1-methylethylidene)-beta-D-fructopyranose sulfamate), has shown effective seizure control in primary generalized tonic-clonic seizures, newly diagnosed epilepsy, refractory focal seizures, and seizures associated with Lennox-Gastaut syndrome.^[10–16]^ In addition, topiramate is almost 3 times more effective in reducing seizures as an add-on treatment for drug-resistant focal epilepsy. Compared with placebo, the relative risk (RR) of topiramate as add-on therapy for >50% seizure reduction was 2.71 (95% CI = 2.05–3.59).^[17,18]^ In a randomized, placebo-controlled study^[19]^ and an open-label, observational study,^[20]^ topiramate was well tolerated at a dosage of 200–600 mg per day and effectively reduced seizure frequency in add-on therapy. In the maintenance phase, 83% of patients had a >50% seizure reduction.^[20]^ Other randomized trials in Korea also showed that the seizure response rates for topiramate were 80% for patients with focal-onset aware motor seizure,^[21]^ 60% for patients with focal-onset impaired awareness seizures,^[21]^ 64.8% for patients with focal epilepsy,^[22]^ and 50.6% for patients with medically intractable focal epilepsy.^[23]^ The results of these clinical trials suggest that topiramate has different levels of efficacy in seizure response rates for different seizure types. However, to date, no studies have investigated the efficacy of topiramate in patients with epilepsy following neurosurgery. Thus, this study investigated the clinical efficacy and safety of dose-escalated topiramate as first line monotherapy and add-on therapy in adult patients with neurosurgery-related epilepsy.

## 2 Materials and methods

### 2.1 Patients

This study was conducted in accordance with the Declaration of Helsinki, and the protocol was approved by the Institutional Review Board of the hospital ethic committee (No. 09CT12049b). Signed informed consent was obtained from all participants. The inclusion criteria were:

1. patients who developed symptoms of epilepsy after neurosurgery;
2. patients aged 18–65 years; and
3. patients who were taking antiepileptic drugs (except for topiramate) at least 4 weeks before entering the trial, but with poor seizure control.

The exclusion criteria were:

1. patients with seizures due to generalized etiology such as metabolism, infection or intoxication;
2. patients with grade IV glioblastoma multiforme or tumors with multiple metastases;
3. patients with a history of psychosis or seizure cluster;
4. patients with a history of alcohol or drug abuse;
5. patients who received topiramate treatment or participated in a clinical trial within 12 weeks before entering the trial;
6. patients with a history of mood disorder who need electroconvulsive therapy or tranquilizers; and
7. patients who cannot take medications or maintain a seizure calendar independently or with the assistance of others.

Finally, a total of 55 eligible patients were recruited from 2010 to 2011, and the patients were randomly divided into topiramate monotherapy and stepwise add-on therapy groups. Because of individual medical histories, 7 patients switched their initial assigned add-on therapy to monotherapy, eventually resulting in 35 patients in the monotherapy group and 20 patients in the additional therapy group. Since all patients participated in this study within 6 years after surgery, the duration of epilepsy in the 2 groups was 5.0 years and 5.9 years for topiramate monotherapy and add-on therapy, respectively.

### 2.2 Trial design

**Figure 1:**
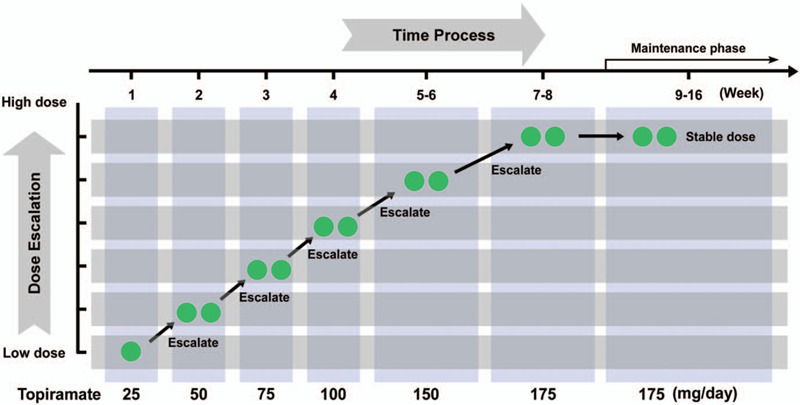
Topiramate dose escalation scheme for neurosurgical patients. An initial dose of 25 mg/day was used in the first week and an increase of 25 mg per week until the fourth week. The topiramate doses at weeks 5–6 and 7–8 were 150 and 175 mg/day, respectively. In the maintenance phase, patients received a fixed 175 mg/day dosage regimen. Except for the first week, the total daily doses for other weeks was divided in to 2 portions for patients (twice-daily dosing).

In this study, the topiramate dose escalation method was used in 2 cohorts of patients, topiramate monotherapy and stepwise add-on therapy. For add-on therapy, valproate and phenytoin were the main anti-seizure medications prior to adding on topiramate. The starting dose of topiramate was 25 mg/day in the first week, and the dose increased weekly by 25 mg to the fourth week (Fig. 1). The topiramate doses at weeks 5–6 and 7–8 were 150 and 175 mg/day, respectively. In the maintenance phase (after the eighth week of treatment), patients received a fixed 175 mg/day dosage regimen. Except for the first week, the total daily doses in the other weeks were divided into 2 portions for patients (twice-daily dosing). In addition to dose escalation of topiramate as described above, patients in the add-on therapy group also continued to take prior anti-epileptic drugs.

### 2.3 Evaluation

During treatment, patients themselves (or other family members who could help with the recording) recorded the frequency and type of seizures daily and followed up monthly to assess adverse events. The efficacy of monotherapy and add-on therapy was analyzed by the seizure-free rate and seizure response rate. Seizure-free rate was defined as the percentage of patients who did not have any seizure episodes. Seizure response rate was defined as the proportion of patients with a reduction in seizure frequency of at least 50% or maintaining a seizure-free status. Adverse events were coded according to the Medical Dictionary for Regulatory Activities (MedDRA),^[14]^ which was used to map verbatim adverse events to preferred terms and system organ class. The incidence of adverse events after monotherapy and add-on therapy was calculated and compared between the 2 treatment groups.

### 2.4 Statistical analysis

All data were statistically analyzed by SAS software version 9.4 (SAS Institute Inc., Cary, NC, USA). According to data distribution, continuous variables of demographic and clinical characteristics are expressed as mean ± standard deviation (SD), and statistical significance was determined using ANOVA. Frequency and percentage statistics were used to represent categorical variables and analyzed using the Fishers exact test. Logistic regression analysis was performed to analyze the seizure-free rate, and Poisson regression model was used to analyze seizure frequency. Relative risk (RR) with 95% confidence intervals (CI) was used as a measure of the associations between different treatments and outcomes. *P* < .05 was considered statistically significant.

## 3 Results

A total of 55 eligible patients were enrolled in this study and were divided into 2 treatment groups receiving alternative monotherapy and add-on therapy. Table 1 lists the general demographics and baseline characteristics of the patients. No significant differences were found between the 2 groups in gender, age, weight, height, body mass index (BMI), duration of epilepsy, etiology, seizure type, and dissatisfaction with prior anti-epileptic drugs. Overall, most patients in both groups were male. The mean age of patients in the alternative monotherapy group and add-on therapy group was 44.1 ± 11.9 and 42.4 ± 12.2 years, respectively. The history of epilepsy in patients treated with topiramate monotherapy and add-on therapy was 5.0 ± 7.2 years and 5.9 ± 5.8 years, respectively. The documented etiology of these patients included trauma, vascular diseases such as arteriovenous malformation (AVM), aneurysm, and tumors such as cavernoma, meningioma and glioma. All patients were dissatisfied with previous anti-epileptic treatment, including dissatisfaction with seizure frequency, prolonged seizures, and intolerable adverse events.

**Table 1:** Demographics and baseline characteristics of enrolled patients.

Characteristics	Monotherapy	Add-on Therapy
Gender		
Male	22 (62.9%)	13 (65.0%)
Female	13 (37.1%)	7 (35.0%)
Age (years)	44.1 ± 11.9	42.4 ± 12.2
Weight (kg)	64.5 ± 12.1	71.5 ± 16.8
Height (cm)	165.2 ± 8.6	166.4 ± 9.3
BMI (kg/m^2^)	23.6 ± 3.8	25.6 ± 4.4
Duration of epilepsy (years)	5.0 ± 7.2	5.9 ± 5.8
Etiology		
Tumors (cavernoma, meningioma, glioma, etc)	17 (48.6%)	5 (25.0%)
Vascular disease (AVM, aneurysm, stroke, etc)	7 (20.0%)	1 (5.0%)
Trauma	8 (22.9%)	7 (35.00%)
Other disease	2 (5.7%)	2 (10.0%)
Seizure type		
Focal Onset Aware Seizures	13 (37.1%)	10 (50.0%)
Focal Onset Impaired Awareness	5 (14.3%)	6 (30.0%)
Focal to Bilateral Tonic Clonic	3 (8.6%)	1 (5.0%)
Generalised Onset motor seizure	13 (37.1%)	6 (30.0%)
Unknown Onset	1 (2.9%)	0 (0.0%)
Unsatisfied reason for prior AED treatment		
Unsatisfied seizure frequency	13 (37.1%)	11 (55.0%)
Prolongation of seizure	2 (5.7%)	2 (10.0%)
Intolerable AE	20 (57.1%)	7 (35.0%)

BMI = body mass index; AE = adverse events; AVM = arteriovenous malformation; AED = anti-epileptic drugs.

Patients in the alternative monotherapy groups received only escalated doses of topiramate, as described in Materials and Methods and Figure 1, while patients in the add-on therapy group received escalated doses of topiramate, valproate and phenytoin. In the first month after treatment, patients in the monotherapy group had a better seizure response rate than patients in the add-on therapy group (89% vs 65%, *P* < .05, Table 2). After 2 months of treatment, however, no significant differences were found in seizure response rates between the 2 groups. The seizure response rates in the second, third, and fourth months of treatment in the monotherapy and add-on therapy groups were 92% vs 94%, 80% vs 86%, and 88% vs 70%, respectively. The mean seizure frequency (per month) in the alternative monotherapy group and add-on therapy group was 0.725 and 0.536, respectively (*P* = .756, Table 2,). Furthermore, the seizure-free rates in monotherapy and add-on therapy groups were 88.0% and 78.6%, respectively. However, no significant differences were found between the 2 groups (*P* = .647), suggesting that the efficacy of topiramate monotherapy was almost the same as that of add-on therapy.

**Table 2:** Seizure frequency and seizure-free response after monotherapy or add-on therapy.

	Monotherapy	Add-on Therapy
Seizure response rate		
1st month	89%	65%**∗**
2nd month	92%	94%
3rd month	80%	86%
4th month	88%	79%
Total seizure count	17	7
Relative risk (95% CI)	1.3065 (0.2414–7.0719)^ns^	
Seizure-free rate	88.0%	78.6%
Difference (95% CI)	9.4% (−22.6%–40.4%)^ns^	

Seizure frequency was analyzed by Poisson regression model. Asterisk denotes significant differences (*P* < .05), and ns indicates non-significant differences.

**Figure 2:**
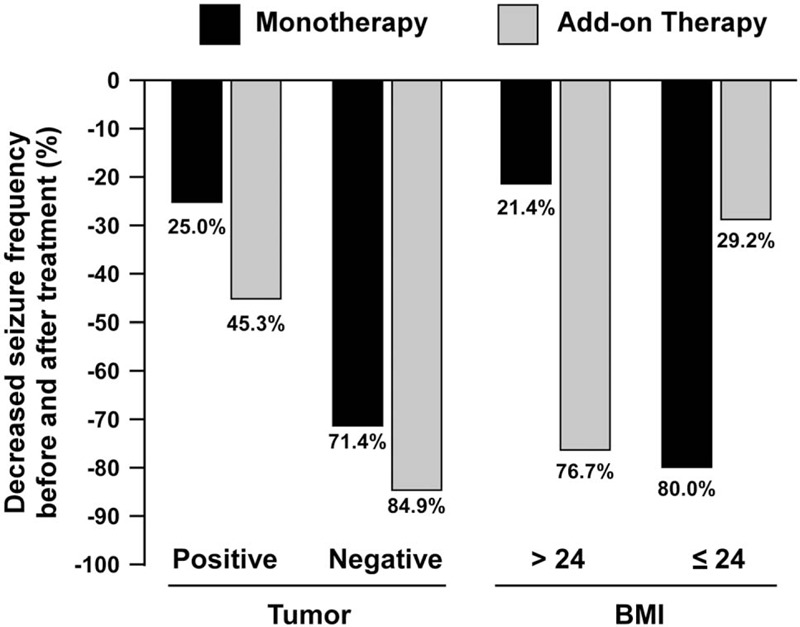
Improved seizure frequency after topiramate monotherapy and add-on therapy treatment. Comparison of decreased seizure frequency in neurosurgical patients with/without tumor (A) and with different baseline BMI (B).

Since epilepsy in cancer patients is usually resistant to anti-epileptic drugs^[24]^ and epilepsy treatment may be associated with substantial weight changes,^[25]^ we analyzed the effects of tumor and BMI on the efficacy of monotherapy and add-on therapy. As shown in Figure 2, patients with tumors showed less improvement in seizure frequency in both the monotherapy and add-on therapy groups. Moreover, both treatments showed good improvement of seizure frequency for patients without tumors. On the other hand, in patients with BMI > 24, add-on therapy showed better efficacy in improving seizure frequency than monotherapy treatment (76.7% vs 21.4%), while the efficacy of add-on therapy was poorer than that of monotherapy (29.2% vs 80.0%) in patients with BMI ≤ 24.

After monotherapy or add-on therapy, most patients experienced adverse events, including dizziness, headache, decreased appetite, somnolence, weight loss, insomnia, pruritus, malaise, nausea, muscular weakness, rhinitis allergic, hyperhidrosis, and rash (Table 3). Among these, dizziness and headache were the most common adverse events in patients in the monotherapy and add-on treatment groups. Except for decreased appetite and weight loss, the occurrence of other adverse events did not differ significantly between the 2 groups. The occurrence of decreased appetite (14.3% vs 5.0%) and weight loss (11.4% vs 5.0%) in the monotherapy group was slightly higher than those in the add-on therapy group.

**Table 3:** Incidence of adverse events after monotherapy or add-on therapy.

Adverse Events	Monotherapy	Add-on Therapy
Dizziness	10 (28.6%)	4 (20.0%)
Headache	6 (17.1%)	3 (15.0%)
Decreased appetite	5 (14.3%)	1 (5.0%)
Somnolence	4 (11.4%)	2 (10.0%)
Weight loss	4 (11.4%)	1 (5.0%)
Insomnia	3 (8.6%)	2 (10.0%)
Pruritus	3 (8.6%)	2 (10.0%)
Malaise	3 (8.6%)	1 (5.0%)
Nausea	3 (8.6%)	0 (0.0%)
Muscular weakness	2 (5.7%)	1 (5.0%)
Rhinitis allergic	3 (8.6%)	0 (0.0%)
Hyperhidrosis	1 (2.9%)	2 (10.0%)
Rash	3 (8.6%)	0 (0.0%)

## 4 Discussion

This study provides clinical evidence of the safety and efficacy of dose-escalated topiramate as monotherapy or add-on therapy for neurosurgical patients with seizure disorder. Comparison of the monotherapy and add-on therapy reported herein demonstrate that topiramate dose escalation to 175 mg/day in the final maintenance stage was still safe and effective in reducing seizure frequency in neurosurgical patients. Both monotherapy and add-on therapy effectively improved the seizure frequency in patients without tumors, but the topiramate dose still needs to be modified to improve epilepsy in tumor patients. It is worth noting that monotherapy is more effective than add-on therapy in patients with BMI ≤ 24. Although most patients experienced adverse events after monotherapy or add-on therapy, no severe adverse events were observed in both therapies, and no significant differences were found in the occurrence of adverse events between the 2 therapies. Thus, topiramate dose-escalation is safe and effective as initial or early monotherapy in neurosurgical patients, and appears to be well-tolerated in both monotherapy and add-on therapy.

Since no statistically significant differences were found in seizure response rates, total seizure counts and seizure-free rates between patients in the monotherapy and add-on therapy groups, that is, topiramate monotherapy was not better than add-on therapy in improving of neurosurgery-related seizures (Table 2). A possible explanation for this result is that the topiramate dose used in this study was lower than the clinically recommended dose. In general, the recommended dose range for topiramate add-on therapy is 200–400 mg/day, while for monotherapy it is 400 mg/day.^[1,16,26]^ A study by Gupta et al^[27]^ showed that increasing the dose of topiramate from 100 mg/day to 200 mg/day increased the seizure response rate from 68.8% to 87.5%. Consistently, 237 mg/day of topiramate ^[20]^ for 12 weeks and 298 mg/day of topiramate ^[28]^ for 6 months had 83% and 82.6 seizure response rates, respectively. In the present study, using a lower dose of topiramate (175 mg/day) produced a 79% seizure response rate. However, increasing the topiramate dosage further did not significantly increase the expected efficacy, but usually increased with the occurrence of adverse reactions.^[29–31]^ When topiramate dosage exceeds 200 mg/day, the seizure response rate gradually decreases.^[27]^ In that study, the seizure response rates in patients taking 200 mg/day, 300 mg/day, and 400 mg/day were 87.5%, 85.7%, and 66.7%, respectively. In a randomized, double-blind, placebo-controlled trial,^[23]^ topiramate tolerance in Asian populations was significantly different from that in Western populations, with average daily doses lower than the recommended 200 mg of topiramate. In the present study, most patients could only tolerate a topiramate dose of up to 175 mg/day. In addition, the Asian population is smaller on average in individual body weight than Westerners, suggesting that the use of lower final doses and dose escalation schedules for Asian populations may lead to better tolerability.^[32–34]^ Previous trials have also demonstrated that efficacy has not improved significantly in patients receiving high doses of topiramate (200 mg/day, 200–400 mg/day, and 400–600 mg/day).^[23,29,30]^ Therefore, patients who have reached acceptable seizure control at the end of the titration period should maintain this maximum dose, since there is no clinical rationale to increase the dose to the recommended range (200–400 mg/day). Furthermore, some trials reported that approximately 20% of patients withdrew from the trial early due to severe adverse events caused by high doses of topiramate at 400 mg/day.^[30,35]^ In addition, for patients with new-onset seizures, the recommended target topiramate monotherapy dose is 100 mg/day, and the optimal dose for add-on therapy does not exceed 400 mg/day.^[36]^ Therefore, a maximum maintenance dose of 175 mg/day in the present trial is reasonable.

Topiramate has been approved for monotherapy and adjunctive therapy in patients with focal-onset seizures or patients with generalized onset motor seizures.^[36]^ In the present study, the final seizure-free rates of topiramate monotherapy and add-on therapy were 88.0% and 78.6%, respectively. The seizure response rates of monotherapy and add-on therapy were 88% and 79%, respectively. The efficacy of monotherapy and add-on therapy with the final maintenance dose of 175 mg/day was comparable to the results of previous randomized controlled trials. A pooled analysis of 6 randomized controlled trials using topiramate as add-on therapy showed that the seizure response rate and seizure-free rate were 43% and 5%, respectively.^[37]^ The topiramate placebo-controlled dose-ranging trial by Faught et al showed that the response rate was 27% for topiramate 200 mg/day.^[30]^ In the open-label, observational study by Krakow et al, the seizure response rate and seizure-free rate of patients in the maintenance phase were 83% and 18%, respectively.^[20]^ In the topiramate add-on therapy for the treatment of refractory focal-onset seizures, the response rate was 80% to 85% during the first 3 months of treatment.^[28]^ Compared to these previous trials, the use of dose escalation of topiramate monotherapy and add-on therapy in the present study were effective in patients with neurosurgical-related seizures. In addition, clinical effectiveness of monotherapy and add-on therapy was evaluated by either the global investigators assessment or global subjects assessment and demonstrated a good improvement rate (data not shown).

Monotherapy has been suggested to be associated with fewer seizures and higher adherence rates.^[38]^ In addition, pharmacologic treatment for epilepsy is associated with substantial weight gain and loss, which may impair adherence to the treatment regimen.^[39]^ Interestingly, unlike other anti-epileptic drugs such as valproate and gabapentin, topiramate is the anti-epileptic drug most associated with weight loss.^[25]^ In an uncontrolled, prospective clinical trial by Ben-Menachem et al.,^[40]^ weight loss was apparent in refractory epilepsy patients with BMI < 25 and BMI ≥ 30. Thus, we analyzed whether the BMI of neurosurgical patients affects the efficacy of topiramate monotherapy and add-on therapy. In contrast to other results, our study found that topiramate monotherapy shows better improvement of seizure frequency in neurosurgical patients with BMI ≤ 24, while topiramate add-on therapy is more effective for patients with BMI > 24. Although it is not clear whether it is related to drug adherence, the results indicate that topiramate monotherapy and add-on therapy do have different effects on different BMI levels. Further prospective study is warranted to clarify this issue.

Since lower topiramate doses and dose escalation strategy were used in this study, most adverse events were mild and well-tolerated. Compared to the study conducted in the Epilepsy Centre Kempenhaeghe, almost 70% of patients discontinued 200 mg/day of topiramate, mainly due to intolerance to adverse events.^[41]^ The occurrence of cognitive impairment is a common severe adverse event,^[41]^ but was not observed in the present study. A lower starting dose (25 mg/day), a slow dose increase (25 mg/week), and a lower maximum dose of topiramate may help to explain this result.^[42]^

The present study has several strengths and limitations. The major strength is the use of prospective study design to recruit neurosurgical patients and to use a dose escalation strategy. The main shortcomings of this study include the open-label design, smaller sample size, and the uncontrolled drug choice. Thus, this study cannot further compare the effects of the topiramate and topiramate-combined different anti-epileptic drugs. However, this study was a pragmatic, prospective investigation that initially focused on understanding the efficacy and safety of escalating dosage of topiramate in monotherapy and add-on therapy for neurosurgery-related seizures. In addition to the larger sample size that should be included in future studies, a comparison of the efficacy of combining different anti-epileptic drugs should be explored. In addition to the indications of adverse events, future research should explore patients quality of life more deeply to help ensure beneficial treatment outcomes.

## 5 Conclusions

In conclusion, a dose-escalation strategy to a final 175 mg/day for topiramate monotherapy and add-on therapy has shown good efficacy and safety with fewer adverse events in improving seizure control in neurosurgical patients. Topiramate monotherapy demonstrates a higher seizure response rate and better improvement of seizure frequency in neurosurgical patients with BMI ≤ 24. This straightforward topiramate dosing and titration schedule allows neurosurgical patients with seizures to benefit from improved seizure control. In addition, for neurosurgical patients with different levels of BMI, different topiramate therapies can be considered to improve the efficacy.

## Acknowledgments

We would like to thank Convergence CT for providing English editing to improve English grammar and language usage.

## Author contributions

**Conceptualization:** Yu-Tse Liu, Guo-Tai Chen, Yin-Cheng Huang, Jih-Tsun Ho, Cheng-Chi Lee, Chen-Nen Chang, Cheng-Chia Tsai.

**Data curation:** Yin-Cheng Huang.

**Formal analysis:** Yin-Cheng Huang.

**Supervision:** Chen-Nen Chang.

**Resources (patient treatment):** Cheng-Chia Tsai

**Writing – original draft:** Yu-Tse Liu, Guo-Tai Chen, Yin-Cheng Huang, Jih-Tsun Ho, Cheng-Chi Lee, Chen-Nen Chang.

**Writing – review & editing:** Yu-Tse Liu, Guo-Tai Chen, Yin-Cheng Huang, Jih-Tsun Ho, Cheng-Chi Lee, Chen-Nen Chang, Cheng-Chia Tsai.
