# Effectiveness of dose-escalated topiramate monotherapy and add-on therapy in neurosurgery-related epilepsy: A prospective study: Erratum

**DOI:** 10.1097/MD.0000000000024582

**Published:** 2021-01-29

In the article, “Effectiveness of dose-escalated topiramate monotherapy and add-on therapy in neurosurgery-related epilepsy: A prospective study”,^[1]^ which appears in Volume 99, Issue 52, Dr. Cheng-Chia Tsai has been added to the article.
